# Material-Device-Circuit Co-optimization of 2D Material based FETs for Ultra-Scaled Technology Nodes

**DOI:** 10.1038/s41598-017-04055-3

**Published:** 2017-07-10

**Authors:** Tarun kumar Agarwal, Bart Soree, Iuliana Radu, Praveen Raghavan, Giuseppe Iannaccone, Gianluca Fiori, Wim Dehaene, Marc Heyns

**Affiliations:** 1 imec, Leuven, Belgium; 2 KUL, Leuven, Belgium; 3 University of Pisa, Pisa, Italy

## Abstract

Two-dimensional (2D) material based FETs are being considered for future technology nodes and high performance logic applications. However, a comprehensive assessment of 2D material based FETs has been lacking for high performance logic applications considering appropriate system level figure-of-merits (FOMs) e.g. delay, and energy-delay product. In this paper, we present guidelines for 2D material based FETs to meet sub-10 nm high performance logic requirements focusing on material requirement, device design, energy-delay optimization for the first time. We show the need for 2D materials with smaller effective mass in the transport direction and anisotropicity to meet the performance requirement for future technology nodes. We present novel device designs with one such 2D material (monolayer black-phosphorus) to keep Moore’s alive for the HP logic in sub-5 nm gate length regime. With these device proposals we show that below 5 nm gate lengths 2D electrostatistics arising from gate stack design becomes more of a challenge than direct source-to-drain tunneling for 2D material-based FETs. Therefore, it is challenging to meet both delay and energy-delay requirement in sub-5 nm gate length regime without scaling both supply voltage (*V*_*DD*_) and effective-oxide-thickness (EOT) below 0.5 V and 0.5 nm respectively.

## Introduction

To keep Moore’s law alive, silicon based tri-gate FinFETs are being used for high performance logic at current technology nodes. With each technology generation, these devices achieve 15% boost in ON current, 50% reduction in energy-delay product, and 0.5x area scaling^1, 2^. To further continue this trend, alternative channel materials e.g. SiGe, Ge, III-V, and novel device architectures e.g. gate-all-around nanowire (NW) FETs are being explored for future technology nodes. III-V materials due to its lower effective mass and electron-phonon scattering promise higher mobilities, thus higher ON currents for logic applications. But, the lower effective mass also poses challenges such as losing control on electrostatistics with the scaling of channel length, and lower charge concentrations owing to limited density-of-states (DOS)^3^. Device architectures such as gate-all-around (GAA) FETs promise to achieve better electrostatistics at scaled gate lengths.

Alternatively, 2D materials are considered for high performance logic roadmap due to their atomic thickness, which offer better scalability in comparison to Si and III–V channel FETs^4^. Within the 2D materials family, monolayer black phosphorus based FET has recently gained popularity as a promising high-performance (HP) logic device option at the end of the semiconductor roadmap due to its superior transport properties^5^. Monolayer (ML) BP shows anisotropic properties such as lower effective mass in armchair direction and 8x higher effective mass in zigzag direction. By aligning the ML BP channel length in armchair direction and channel width in zigzag direction we can achieve higher carrier velocity (mobility) and higher density-of-states (i.e. inversion charge density) respectively, which can effectively result in higher on-state currents. With full-band dissipative simulations, currents in monolayer black phosphorus (ML BP) FETs are reported to be significantly higher than other ML TMD based FETs^6, 7^. The dissipative current in ML BP FET is shown to be around 90% of the ballistic current for 10 nm gate length. Having said that, the lower effective mass in the transport direction pose challenges in maintaining good sub-threshold slope below 10 nm gate lengths in ML BP FET in comparison to other TMD based FETs. Moreover, the efforts to have the stable BP under ambient condition are ongoing^8, 9^. Nevertheless, to evaluate real potential of such materials for high performance logic in sub-10 nm technology nodes, we need to co-optimize material and different device designs to achieve the required circuit-level metrics such as delay, and energy-delay product.

In this paper, using quantum transport simulations of monolayer 2D materials-based-FETs, we analyze the cause of such performance degradation in sub-10 nm gate length regime due to both material and device parameters. We propose device structures with ML BP to enable scaling of gate length in sub-5 nm regime. Further, for a given technology node, we show the selection of supply voltage (*V*_*DD*_) to achieve the required delay and an optimum energy-delay product (EDP).

## Results and Discussion

### Circuit-Level Requirements

In order to benchmark the 2D FETs at the circuit level and understand the energy-delay tradeoff, we choose delay and energy per operation as circuit level figure-of-merits (FOMs). We estimate these circuit level metrics for a simplified version of critical path in CMOS logic, with a CMOS inverter chain and balanced 2D FETs for both p- and n-type transistors. The first-order equations for delay and energy per operation can be written as ref. 10:

1 $${\tau }_{CP}=\frac{{C}_{node}{V}_{DD}}{{I}_{ON}}\cdot {L}_{D};\quad {E}_{tot}={C}_{tot}{V}_{DD}^{2}(\alpha +{L}_{D}\frac{{I}_{OFF}}{{I}_{ON}})$$

where *τ*_*CP*_ is the delay of the critical path with a logic depth *L*_*D*_ and the total capacitance of each node *C*_*node*_. Total energy (*E*_*tot*_) per operation can be written as sum of dynamic and leakage energy. Here, *α*, and *C*_*tot*_ denote the activity factor and the total capacitance of the logic design respectively.

Further, we normalize the total energy and delay by the capacitance of the chip, which is reasonable for the sub-10 nm technology nodes, when the total capacitance is dominated by interconnect capacitances instead of intrinsic device capacitance, given as:

2 $$N{\tau }_{CP}=\frac{{V}_{DD}}{{I}_{ON}}\cdot {L}_{D};\quad N{E}_{tot}={V}_{DD}^{2}(\alpha +{L}_{D}\frac{{I}_{OFF}}{{I}_{ON}});\quad NE\tau ={E}_{tot}\cdot {\tau }_{CP}$$

here *Nτ*_*CP*_, and *NE*_*tot*_ denote the normalized delay and total energy per operation respectively, while the energy-delay product (*NEτ*) signifies that energy and speed are equally weighed for an optimized logic design.

**Figure 1:**
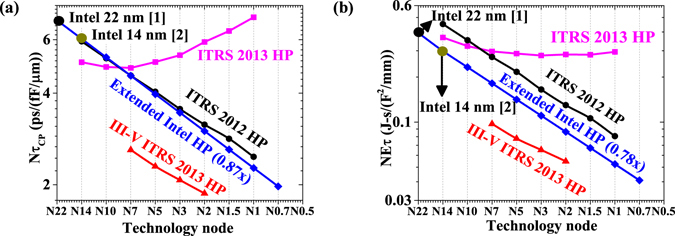
High-performance logic roadmap, (**a**) Normalized delay (*Nτ*_*CP*_) and, (**b**) Normalized energy-delay product (*NEτ*) with technology nodes for *α* = 0.1, and *L*_*D*_ = 10 (HP logic).

As shown in Fig. 1, we extend the circuit-level high performance logic roadmap for sub-5 nm technology nodes by extrapolating scaling of normalized delay and energy-delay product from Intel 22 nm^1^ to Intel 14 nm technology nodes^2^ with reported 15% boost in ON current (for same supply voltage) and required 50% reduction in the energy-delay product. Thus, the scaling of normalized delay by 0.87x and normalized energy-delay product by 0.78x results in total capacitance scaling of around 0.8x with each technology node. Figure 1 shows that extended Intel HP requirements seems most reasonable in comparison to ITRS HP requirements while III-V ITRS HP requirements are quite ambitious.

### Technology Requirements

To achieve area scaling of 0.5x with each technology node, the technology parameters such as contacted gate pitch (*C*_*GP*_) and metal pitch (MP) are scaled by 0.7x with each technology generation. To scale *C*_*GP*_, gate length (*L*_*G*_) scaling has been the primary driver for past technology generations. But, due to process constraints, scaling of *C*_*GP*_ below 25 nm is not forseen^11^. Therefore, for future technology nodes it is imperative to scale gate lengths in sub-10 nm to relax constraints on spacer thickness and contact openings. Alternatively, technology options such as monolithic 3D integration are sought to further scale the area per function^12^. The technology parameters listed in Table 1 (till N2) are taken from III-V ITRS HP roadmap^13^.

**Table 1:** III–V ITRS 2013 HP Roadmap extended for gate lengths below 5 nm.

	N7	N5	N3	N2	N1.5	N1	N0.7
*C*_*GP*_ (nm)	42	32	25	?	?	?	?
*L*_*G*_ (nm)	11.7	9.3	7.4	5.8	4.5	3.5	2.7
*V*_*DD*_ (V)	0.61	0.58	0.56	0.54	?	?	?
*EOT* (nm)	0.62	0.56	0.5	0.45	?	?	?
*I*_*OFF*_ (A/m)	0.1	0.1	0.1	0.1	0.1	0.1	0.1

Supply voltage is denoted by *V*_*DD*_. EOT denotes effective-oxide-thickness. Here, OFF current target (*I*_*OFF*_) for HP logic is chosen to be 100 nA/*μ*m.

### Device-Level Figure-of-Merits

**Figure 2:**
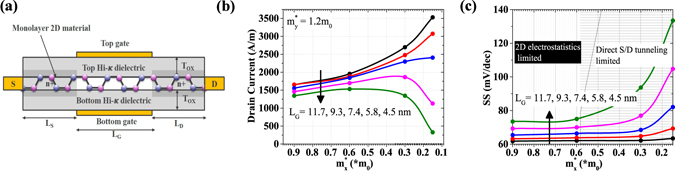
Device Simulations, (**a**) Double-gate monolayer 2D material based FET with *L*_*S*_ = *L*_*D*_ = 15 nm, and *n* ^+^ doping of source/drain = 4 × 10^13^ *cm* ^−2^, and for a fixed transverse effective mass (${m}_{y}^{\ast }$) effect of transport direction effective mass (${m}_{x}^{\ast }$) and gate length (*L*_*G*_) on, (**b**) ON current including effect of scattering and contact resistances, and, (**c**) Extracted subthreshold slope near OFF state.

We consider a double-gate monolayer 2D material based FET as shown in Fig. 2a. The electrical characteristics of 2D material based FETs are calculated using the framework described in methods section. The effect of different transport effective mass and channel lengths on ON current (*I*_*ON*_) is shown in Fig. 2b. We can clearly see that a smaller effective mass 2D material is the preferred choice for high performance logic. Smaller transport mass 2D materials with anisotropic properties can offer higher carrier injection velocity and higher inversion charge density, resulting in higher on-state current provided we can maintain good electrostatistics with gate length scaling. To get physical insights in electrostatics of shorter gate length devices, we study the effect of transport effective mass on sub-threshold slope (S.S.) behavior for different gate lengths as shown in Fig. 2c. We break-down Fig. 2c into two regions: 1) At lower effective masses S.S. degrades due to direct S/D tunneling (due to material property); 2) At higher effective masses where the increase in S.S. with downscaling of gate lengths is attributed to 2D electrostatistics.

### Circuit-Level Figure-of-Merits

**Figure 3:**
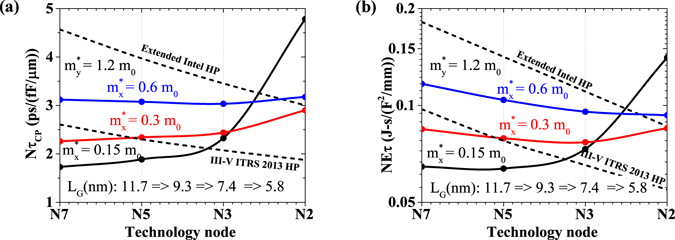
Performance of DG monolayer 2D FET at different technology nodes (**a**) Normalized delay and (**b**) Energy-delay product, showing the effect of gate length scaling of a smaller effective mass material such as monolayer black phosphorus (BP) (${m}_{x}^{\ast }$ = 0.15 *m*_0_, ${m}_{y}^{\ast }$ = 1.2 *m*_0_) w.r.t. III–V ITRS 2013 HP and Extended Intel HP requirements.

Figure 3 shows the combined effect of sub-threshold and super-threshold behavior on delay and energy-delay product for a given *I*_*OFF*_ of 100 nA/*μ*m. We observe that till N3, 2D materials with smaller transport effective mass outperform the 2D materials with higher ones. It can be also seen that monolayer BP (${m}_{x}^{\ast }$ = 0.15 *m*_0_, ${m}_{y}^{\ast }$ = 1.2 *m*_0_) FET can meet both extended Intel HP and III-V ITRS 2013 HP delay and energy-delay requirements for N7, and N5.

### Proposed Device Structures

To further enable the HP logic roadmap with ML BP FETs, we need to improve electrostatistics for sub-10 nm channel lengths with novel device designs. We propose device designs which address improving both 2D electrostatistics and direct source-to-drain (S/D) tunneling.

#### Improving 2-D Electrostatistics

**Figure 4:**
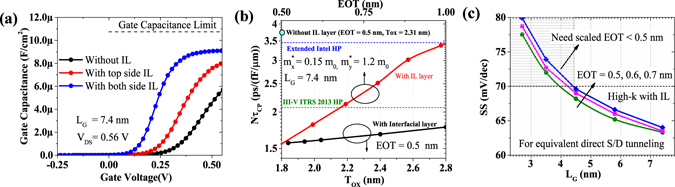
Effect of an interfacial layer (IL) with High-k dielectric, (**a**) On effective gate capacitance with gate voltage, (**b**) On performance (delay) of monolayer BP for N3 technology node i.e. *L*_*G*_ = 7.4 nm in two different cases (i) with different EOT, and (ii) with varying physical oxide thickness for same EOT, (**c**) On the scalability of such gate-stack for different EOT considering equivalent direct source/drain tunneling.

We introduce a low-k interfacial layer (IL) between ML BP and High-k dielectric to reduce fringing fields due to the gate stack at shorter gate lengths^14^. As shown in Fig. 4a, by reducing fringing fields we improve both the gate control (i.e. slope of gate capacitance with gate voltage) and effective gate capacitance in ON state. Further, Fig. 4b shows that the performance of ML BP FETs at *L*_*G*_ = 7.4 nm improves by more than 50% for the same effective-oxide-thickness (EOT) and physical thickness of the gate oxide (*T*_*OX*_). For effective-oxide-thicknesses above 0.5 nm, we consider low-*κ* IL (*SiO*_2_) to be between 0.4–0.6 nm and High-*κ* dielectric (*HfO*_2_, *ZrO*_2_, *La*_2_ *O*_3_) to be 1–1.5 nm thick. To meet both extended Intel HP and III-V ITRS 2013 HP delay requirement with the device structure having low-k IL, we can relax EOT requirements of the N3 technology node. Further, to see prospects of such gate stack with gate length scaling, we consider equivalent direct S/D tunneling probability i.e. ~exp (−*L*_*G*_ · ${\sqrt{m}}_{x}^{\ast }$) as shown in Fig. 4c. It shows that to achieve reasonable 2D electrostatistics below 4.5 nm gate length, we require EOT scaling below 0.5 nm irrespective of the direct S/D tunneling.

#### Reducing Direct Source-to-Drain Tunneling

**Figure 5:**
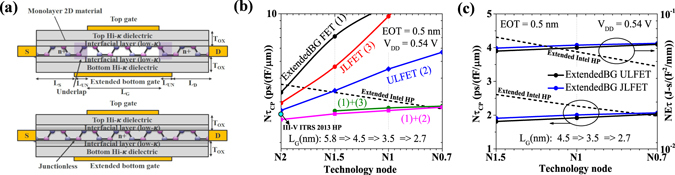
Reducing direct S/D tunneling for N2 and beyond (**a**) Device structures showing extended back-gate (BG) with underlap (ULFET) and extended BG with junctionless doping profile. (JLFET) (**b**) Effect of different combination of device structures on performance of monolayer BP FET for N2 and beyond for fixed EOT and supply voltage, showing the need of extended back-gate with UL/JLFET for *L*_*G*_ = 4.5 nm and beyond, (**c**) Delay and energy-delay product for extended BG UL/JFET (*L*_*UN*_ = 2,3,4 nm for N1.5, N1, and N0.7 respectively) showing that although we meet the performance requirement for N1 and N0.7, the energy-delay product doesn’t scale.

We consider different device concepts (as shown in Fig. 5a) which employ depletion at the source/drain extension-to-channel junction in OFF state, resulting in larger tunneling lengths by modifying the potential profile at the junctions. Although, underlap (UL) and junctionless (JL) 2D material based FETs have been shown to improve direct source-to-drain tunneling at scaled gate lengths^15^, such designs alone can’t provide required performance below 5 nm gate lengths as shown in Fig. 5b. To achieve the required performance for sub-5 nm gate lengths, we propose extended back-gate device architecture in conjunction with UL/JL FET, which makes it possible to meet the performance requirements till N0.7 (*L*_*G*_ = 2.7 nm) for a fixed *V*_*DD*_, and EOT. It is important to note that due to back-gate overlap in the extended back-gate architecture, an extra parasitic capacitance component as gate overlap capacitance comes in picture which may affect the total capacitance scaling, thus delay and energy-delay scaling. Nevertheless, Fig. 5c shows the need to scale *V*_*DD*_ to meet energy-delay requirement although the performance (delay) requirement is met till N0.7 for a fixed *V*_*DD*_.

### Energy-Delay Optimization

**Figure 6:**
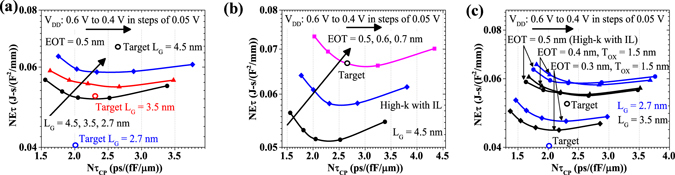
Supply voltage for optimum energy delay product, (**a**) Effect of supply voltage on energy and delay for fixed EOT = 0.5 nm, and *L*_*G*_ = 3.5, and 2.7 nm. (**b**) Effect of EOT scaling on energy-delay Vs delay plot for *L*_*G*_ = 4.5 nm, (**c**) For *L*_*G*_ = 3.5 nm, and *L*_*G*_ = 2.7 nm.

As shown in Fig. 6a, it is very challenging to meet both energy-delay and delay requirement even for smaller supply voltages for N1.5 and beyond. On the other hand, we see that the EOT requirement for N1.5 can be relaxed as shown in Fig. 6b, while Fig. 6c shows that we need to scale EOT below 0.5 nm to meet N1 requirements which scales the supply voltage. As EOTs below 0.5 nm become challenging to achieve using High-k dielectric with IL layer; it requires the advent of two-dimensional oxides with higher dielectric constant, and higher tunneling barrier with ML BP.

### Effect of contact resistance and scattering

**Figure 7:**
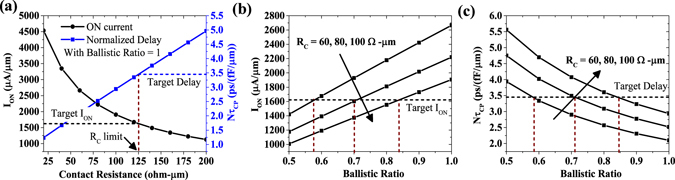
For N3 node with optimized device, (**a**) Effect of S/D contact resistance on ballistic ON current and delay, (**b**) Effect of ballistic ratio on ON current, (**c**) Delay, for different contact resistance values.

Lastly, to understand the limit on different contact resistances and different ballitsic ratios, we first optimize the device structure consisting of High-*κ* with IL and extended back-gate with underlap for technology node N3. The device parameters are taken from Table 1 and the optimized *L*_*UN*_ comes out to be 1 nm. As shown in Fig. 7a, both *I*_*ON*_ and *Nτ*_*CP*_ degrades by increasing contact resistance (*R*_*C*_). We notice the upper limit of contact resistance to be 125 Ω-*μ*m considering no scattering in the channel. Further, Fig. 7b,c show that for *R*_*C*_, ranging between 60 to 100 Ω-*μ*m, we need to have ballisticity in the channel material between 85% to 60% respectively.

## Conclusions

In this paper, we show that monolayer black phosphorus based FETs with different device designs can fulfill the high-performance logic energy-delay requirements till sub-5 nm gate lengths. Although the monolayer black phosphorus is reported to be unstable under ambient conditions and efforts to have the stable BP are ongoing, we infer that lower transport effective mass 2D material such as monolayer BP (with proposed device designs) perform better than higher effective mass 2D materials. To boost the performance of 2D material FET for advanced technology nodes, we propose device structures consisting of High-*κ* with IL (to increase the effective device gate capacitance), and extended back-gate with underlap (to curb direct source-to-drain tunneling). To meet the HP logic requirements, Table 2 lists the choice of device structure, and technology/device/circuit level parameters such as EOT/*I*_*ON*_/*V*_*DD*_. We see that for N1 and beyond, scaling of *V*_*DD*_ below 0.5 V becomes increasingly hard in order to meet both delay and energy-delay requirements, due to 60 mV/dec sub-threshold slope limit of FETs. It instigates the requirement of sleep sub-threshold slope transistors with effective ON currents ~2000 *μ*A/*μ*m.

**Table 2:** Technology/device/circuit level parameters for high performance logic roadmap, and monolayer black phosphorus device architectures: (1) Double gate + High-k dielectric with IL, (2) Double gate + High-k dielectric with IL + Underlap, (3) Double gate + High-k dielectric with IL + extended BG + UL/JL, (4) Double gate + thinner 2D high-k oxide + extended BG + UL/JL.

	N7	N5	N3	N2	N1.5	N1	N0.7
*C*_*GP*_ (nm)	42	32	25	?	?	?	?
*L*_*G*_ (nm)	11.7	9.3	7.4	5.8	4.5	3.5	2.7
*V*_*DD*_ (V)	0.61	0.58	0.56	0.54	0.5	0.47	0.45
*EOT* (nm)	>1	>1	~1	~0.8	~0.7	0.35	0.3
Device architecture	1	1	1	2	3	4	4
*I*_*ON*_ (A/m)	…	…	1657.5	1855.4	1939.7	2098	1961.5
*I*_*OFF*_ (A/m)	0.1	0.1	0.1	0.1	0.1	0.1	0.1

Here, ON current (*I*_*ON*_) includes the effect of contact resistance and scattering.

## Methods

The electrical characteristics of 2D material based FETs in the ballistic limit are calculated using a two-band tight binding (TB) Hamiltonian with a quantum transport simulation framework based on self-consistent solution of Poisson and Schrödinger equation with non-equilibrium Green’s function within the NanoTCAD ViDES suite^16^. The two-band Hamiltonian for an anisotropic effective mass two-dimensional material with hexagonal lattice can be written as a 2 × 2 Hamiltonian matrix:

3 $${H}_{2D}=[\begin{array}{cc}{E}_{cm} & f(k)\\ {f}^{\ast }(k) & {E}_{vm}\end{array}]$$

where *E*_*cm*_, and *E*_*νm*_ denote the bottom of conduction band, and top of the valence band. Further, bandgap (*E*_*G*_) of the material can be expressed as: *E*_*G*_ = *E*_*cm*_ − *E*_*νm*_. Here, the *f*(*k*) function, due to nearest neighbors, can be written as:

4 $$f(k)={t}_{1}{e}^{(-i{k}_{x}a/\sqrt{3})}+2{t}_{2}{e}^{(i{k}_{x}a\mathrm{/2}\sqrt{3})}\,\cos \,(\frac{{k}_{y}a}{2}).$$

Here, *t*_1_, *t*_2_ represents hopping energies in x and y direction respectively, which are calculated using the effective masses in x and y directions and bandgap of 2D material. *k*_*x*_, and *k*_*y*_ are wave vectors in x & y directions, while *a* denotes the lattice constant of the two-dimensional hexagonal lattice. Further, using secular equation, we obtain the dispersion relation for the two-band model given as:

5 $${E}^{\pm }(k)=\frac{({E}_{cm}+{E}_{vm})\pm \sqrt{{({E}_{cm}-{E}_{vm})}^{2}+4{|f(k)|}^{2}}}{2}.$$

In order to calculate *t*_1_ and *t*_2_ for given effective masses in x and y direction, we use the parabolic effective mass approximation with the two-band model as:

6 $$\frac{1}{{m}_{x}^{\ast }}=\frac{1}{{\hslash }^{2}}\cdot \frac{{\partial }^{2}{E}^{\pm }(k)}{\partial {k}_{x}^{2}}\quad \frac{1}{{m}_{y}^{\ast }}=\frac{1}{{\hslash }^{2}}\cdot \frac{{\partial }^{2}{E}^{\pm }(k)}{\partial {k}_{y}^{2}}$$

where ${m}_{x}^{\ast }$ and ${m}_{y}^{\ast }$ denotes the reduced effective mass in x and y direction. Using Eqs 4–6, and by taking limit of the second derivative at the minimum energy k-point, we can calculate *t*_1_ and *t*_2_ for a given ${m}_{x}^{\ast }$, ${m}_{y}^{\ast }$, and *E*_*G*_ as:

7 $${|{t}_{1}|}^{2}=\frac{2{\hslash }^{2}{E}_{G}}{3{a}^{2}{m}_{x}^{\ast }}\quad {|{t}_{2}|}^{2}=\frac{{\hslash }^{2}{E}_{G}}{2{a}^{2}}[\frac{1}{3{m}_{x}^{\ast }}+\frac{1}{{m}_{y}^{\ast }}]$$

Further, to calculate practical currents from ballistic currents, first we calibrate our results with full-band dissipative simulations of ML BP FETs at 10.5 nm gate length using monolayer BP material parameters, which results in ballistic ratio of around 0.9 as mentioned in ref. 6. Moreover, the effect of source/drain contact resistances are included according to ITRS guidelines (i.e. linear degradation in the intrinsic ON current from 33% (in 2011) to 40% (in 2026)) to benchmark the performance of intrinsic 2D material based FETs with III-V ITRS HP roadmap^13^. Effectively, degradation of around 44% is considered in the ballistic currents due to scattering and contact resistance for Table 2.
